# The real-world efficacy and safety of faricimab in neovascular age-related macular degeneration: the TRUCKEE study – 6 month results

**DOI:** 10.1038/s41433-023-02553-5

**Published:** 2023-05-12

**Authors:** Arshad M. Khanani, Aamir A. Aziz, Hannah Khan, Ashwin Gupta, Ohidul Mojumder, Aigerim Saulebayeva, Ashkan M. Abbey, David R. P. Almeida, Robert L. Avery, Himanshu K. Banda, Mark R. Barakat, Ramanath Bhandari, Emmanuel Y. Chang, Sara J. Haug, Nikolas J. S. London, Luke Mein, Veeral S. Sheth, Jeremy D. Wolfe, Michael A. Singer, Carl J. Danzig

**Affiliations:** 1 Sierra Eye Associates, Reno, NV USA; 2 University of Nevada, Reno School of Medicine, Reno, NV USA; 3 Vanderbilt University School of Medicine, Nashville, TN USA; 4 Texas Retina Associates, Dallas, TX USA; 5 Erie Retinal Surgery, Erie, PA USA; 6 California Retina Consultants, Santa Barbara, CA USA; 7 Sound Retina, Tacoma, WA USA; 8 Retinal Consultants of Arizona, Phoenix, AZ USA; 9 Springfield Clinic Eye Institute, Springfield, IL USA; 10 Retina & Vitreous of Texas, Houston, TX USA; 11 Southwest Eye Consultants, Durango, CO USA; 12 Retina Consultants San Diego, San Diego, CA USA; 13 Medical Center Ophthalmology Associates, San Antonio, TX USA; 14 University Retina, Chicago, IL USA; 15 Associated Retinal Consultants, Royal Oak, MI USA; 16 Rand Eye Institute, Deerfield Beach, FL USA

**Keywords:** Outcomes research, Macular degeneration

## Abstract

**Background/Objective:**

Investigate real-world patients receiving faricimab for the treatment of neovascular age-related macular degeneration (nAMD).

**Subjects/Methods:**

Multicenter, retrospective chart review was conducted on patients treated with faricimab for nAMD from February 2022 to September 2022. Collected data includes background demographics, treatment history, best-corrected visual acuity (BCVA), anatomic changes, and adverse events as safety markers. The main outcome measures are changes in BCVA, changes in central subfield thickness (CST) and adverse events. Secondary outcome measures included treatment intervals and presence of retinal fluid.

**Results:**

After one injection of faricimab, all eyes (*n* = 376), previously-treated (*n* = 337) and treatment-naïve (*n* = 39) eyes demonstrated a + 1.1 letter (*p* = 0.035), a + 0.7 letter (*p* = 0.196) and a + 4.9 letter (*p* = 0.076) improvement in BCVA, respectively, and a − 31.3 μM (*p* < 0.001), a − 25.3 μM (*p* < 0.001) and a − 84.5 μM (*p* < 0.001) reduction in CST, respectively. After three injections of faricimab, all eyes (*n* = 94), previously-treated (*n* = 81) and treatment-naïve (*n* = 13) eyes demonstrated a + 3.4 letter (*p* = 0.03), a + 2.7 letter (*p* = 0.045) and a + 8.1 letter (*p* = 0.437) improvement in BCVA, and a − 43.4 μM (*p* < 0.001), a − 38.1 μM (*p* < 0.001) and a − 80.1 μM (*p* < 0.204) reduction in CST, respectively. One case of intraocular inflammation was observed after four injections of faricimab and resolved with topical steroids. One case of infectious endophthalmitis was treated with intravitreal antibiotics and resolved.

**Conclusions:**

Faricimab has demonstrated improvement or maintenance of visual acuity for patients with nAMD, along with rapid improvement of anatomical parameters. It has been well-tolerated with low incidence of treatable intraocular inflammation. Future data will continue to investigate faricimab for real-world patients with nAMD.

## Introduction

Neovascular AMD (nAMD), also known as wet or exudative AMD is characterized by the growth of new, abnormal vasculature from the choriocapillaris extending into the retina, threatening the photoreceptors or retinal pigment epithelium (RPE). Exudation, fluid accumulation and haemorrhages from the vessels can result in vision loss, via RPE detachment or subretinal fibrosis if not promptly treated [1].

The introduction of the intravitreal injection of anti-vascular endothelial growth factor (anti-VEGF) agents was a major paradigm shift for the management of patients suffering from nAMD by providing efficacious and safe treatment to preserve vision. Four agents have been subsequently approved by the FDA, pegaptanib (OSI Pharmaceuticals, Long Island, NY, USA), ranibizumab (Genentech Inc, San Francisco, CA, USA), aflibercept (Regeneron, Tarrytown, NJ, USA) and brolucizumab (Novartis, Basel, Switzerland). The off-label use of bevacizumab (Genentech Inc, San Francisco, CA, USA) is also common practice by retinal specialists worldwide [2–5]. Labels for the approved drugs indicate treatment intervals ranging from four to twelve weeks, with patients typically receiving injections every six to seven weeks [6]. This high treatment-burden has been identified as a significant unmet need in the management of nAMD.

As of January 2022, the newest agent to be approved by the FDA is faricimab, a novel bispecific, monoclonal antibody that targets both VEGF-A and angiopoetin-2 (Ang-2). Both pathways are implicated in neovascularization and vascular leakage, but Ang-2 has been noted to further vascular instability along with increase exudation [7]. Simultaneous inhibition of these pathways by the bispecific faricimab was theorized to result in more complete blockage of neovascularization and exudation with improved vascular stability [8].

Positive outcomes in Phase II trials investigating faricimab led to the initiation of the Phase III studies, TENAYA and LUCERNE. These identical, double-masked trials were conducted in over 1300 treatment-naïve nAMD patients globally. The primary endpoint was the change from baseline in best-corrected visual acuity (BCVA) averaged over weeks 40, 44 and 48 (based on cohort). Patients in the faricimab arms in TENAYA and LUCERNE improved +5.8 or +6.6 letters, respectively, while patients in the aflibercept arm improved +5.1 and +6.6 letters, demonstrating non-inferiority [9]. Comparable reductions in central subfield thickness (CST), choroidal neovascularization (CNV) size and leakage were observed for both treatments. Safety was also similar for both faricimab and aflibercept. The durability of faricimab was particularly noteworthy, with nearly 80% of patients in the faricimab arm maintaining either 16- or 12-week dosing intervals in year one data, with 45% achieving a 16-week dosing interval [10]. Year two data found that 60% of patients treated with faricimab could be maintained at 16-week intervals with similar visual gains to Q8W aflibercept, along with comparable anatomical outcomes [11].

These positive results from two Phase III trials led to approval of faricimab for nAMD with a treatment label indicating dose intervals ranging from Q4W to Q16W following four monthly loading doses. However, real-world evidence of faricimab utilization in clinical practice has yet to be published especially in high-need previously-treated patients. These patients are often excluded from registrational studies and the generation of their real-world efficacy and safety data is of particular importance for practicing retinal physicians and other healthcare providers. The TRUCKEE study is a real-world, collaborative study aimed at investigating the efficacy, durability, and safety of faricimab in patients suffering from nAMD in routine clinical practice.

## Methods

### Participants

Patients from 14 sites (Sierra Eye Associates, Reno, NV; Texas Retina Associates, Dallas, TX; Erie Retinal Surgery, Erie, PA; California Retina Consultants, Santa Barbara, CA; Sound Retina, Tacoma, WA; Retinal Consultants of Arizona, Mesa, AZ; Springfield Clinic Eye Institute, Springfield, IL; Retina & Vitreous of Texas, Houston, TX; Rand Eye Institute, Deerfield Beach, FL; Southwest Eye Consultants, Durango, CO; Retina Consultants San Diego, San Diego, CA; University Retina, Chicago, IL; Associated Retinal Consultants, Royal Oak, MI; Medical Center Ophthalmology Associates, San Antonio, TX) were identified based on the inclusion criteria of receiving faricimab for the treatment of nAMD. It was determined by the Advarra Institutional Review Board (IRB) that the study is exempt from IRB oversight as no patient-identifying information is collected. Confidentiality was maintained at individual sites to ensure that no shared data or data aggregate would include any identifying information. All patients who received faricimab for the treatment of nAMD were included and no excluding criteria were placed to additionally filter subjects, making this a true real-world analysis.

### Study design

Inclusion criteria for this study were patients who had received faricimab for the treatment of nAMD post FDA-approval. No exclusion criteria were placed, but only patients with a follow-up visit are included in analysis (some patients had not completed a follow-up at time of data-cut). Collected data included demographics (age, gender), treatment history if applicable (number of previous treatments, type of previous treatment, previous treatment interval, history of uveitis and intraocular inflammation (IOI)), visual acuity, CST, presence of retinal fluid (intra- or subretinal), presence of pigment epithelial detachment (PED) and PED height if applicable, and adverse events. Presence of retinal fluid or PED was determined by each investigator as demonstrated on optical coherence tomography (OCT). PED height was measured via OCT software measurement tool. Snellen visual acuity was converted to the Early Treatment Diabetic Retinopathy Study (ETDRS) BCVA scoring via the formula “ETDRS = 85 + 50 × log_10_ (Snellen Fraction)” [12].

### Statistical analysis

Baseline and demographic characteristics are summarized using descriptive statistics (means for continuous variables such as age and percentages for categorical variables such as previous treatment type). The primary objective of the study is to provide data analysis of the real-world efficacy and safety of intravitreal faricimab for nAMD. Efficacy is reported as changes in quantifiable data, such as BCVA, CST and PED height, along with percentages of patients presenting with binary categorical disease markers, such as presence of PED and intraretinal (IRF) and subretinal fluid (SRF). Efficacy is also subdivided based on previous treatment history, such as prior treatment with anti-VEGF or treatment-naïve, to determine improvements in specific populations. Safety is summarized, presenting the adverse event and treatment. Adverse events are defined as unfavourable events in health, ocular or otherwise, including but not limited to intraocular inflammation, retinal artery occlusion or significant and rapid worsening of vision or anatomy. Significance values were determined using two-tailed t-test analysis, with a significance value of *p* < 0.05 used for all analyses.

## Results

### Demographics

Three hundred and thirty-five patients with 376 eyes had a follow-up visit after one injection of faricimab. Of these, 39 eyes were treatment-naïve and 337 were previously-treated with anti-VEGF, of which 237 received aflibercept at their previous visit. The average age was 79.8 years, with a range from 44–100 years. Fifty-five percent of patients were female. Complete demographics of patients with follow-up after one injection of faricimab are provided in Table 1.

**Table 1:** Demographics of All Patients with Follow-Up after One Faricimab Injection.

Variable	Mean	Range
Age (years)	79.8	44*–100
Variable	Groups	*N* (%)
Gender	Male	150 (44.8%)
	Female	185 (55.2%)
Previous anti-VEGF Agent	Aflibercept	237 (63.0%)
	Ranibizumab	58 (15.4%)
	Brolucizumab	26 (6.9%)
	Bevacizumab	16 (4.3%)
	Treatment-Naïve	39 (10.4%)

This table lists the collected demographics of patients, including age, gender and specific treatment history.

*Of note, a patient aged 44 years was included in analysis, an age well below the expected onset of nAMD. This patient was confirmed to have CNV secondary to Best vitelliform macular dystrophy and was included in the analysis as patients were identified by EMR or billing codes, which includes this secondary CNV. For consistency, the patient was retained in the analysis and results.

### Efficacy

#### Follow-up after one faricimab injection

All patients with a follow-up visit after one injection of faricimab (*N* = 376 eyes) demonstrated a mean BCVA increase of +1.1 letters (*p* = 0.035) and a mean CST reduction of −31.3 μM (*p* < 0.001). Patients switched from any anti-VEGF (*N* = 337 eyes) demonstrated a mean BCVA increase of +0.7 letters (*p* = 0.196) and a mean CST reduction of −25.3 μM (*p* < 0.001), at a similar treatment interval to their previous agent. Previously-treated patients had a mean prior interval of treatment of 44.2 days, with a follow-up interval after one faricimab injection of 43.5 days. Most of these patients were difficult to treat and high-need, with an average of 31.1 injections in their treatment history prior to their first faricimab injection. Patients switched from aflibercept to faricimab (*N* = 237 eyes) demonstrated a mean BCVA increase of +0.2 letters (*p* = 0.782) and a mean CST reduction of −26.3 μM (*p* < 0.001) at a similar treatment interval to their previous agent. Treatment-naïve patients (*N* = 39 eyes) demonstrated a mean BCVA increase of +4.9 letters (*p* = 0.076) and a mean CST reduction of −84.5 μM (*p* < 0.001). Complete data for patient efficacy after one injection of faricimab is provided in Table 2.

**Table 2:** Efficacy after One Injection of Faricimab.

Overall Efficacy (*N* = 335 patients, 376 eyes)				
	Baseline	Follow-Up	Change	*P*-Value
Variable	Mean [SEM]	Mean [SEM]		
ETDRS (letters)	59.5 letters [0.06]	60.6 letters [0.05]	+1.1 letters	0.035
CST (μM)	334.3 μM [0.32]	303.0 μM [0.28]	−31.3 μM	<0.001
PED Height* (μM)	244.5 μM [1.37]	185.6 μM [1.59]	−58.9 μM	<0.001
Efficacy in Patients Switched from Any Anti-VEGF (*N* = 298 patients, 337 eyes)				
	Baseline	Follow-Up	Change	*P*-Value
Variable	Mean [SEM]	Mean [SEM]		
ETDRS (letters)	60.0 letters [0.06]	60.7 letters [0.06]	+0.7 letters	0.196
CST (μM)	328.0 μM [0.35]	302.7 μM [0.35]	−25.3 μM	<0.001
PED Height* (μM)	244.5 μM [1.55]	185.6 μM [1.60]	−58.9 μM	<0.001
Efficacy in Patients Switched from Aflibercept (*N* = 209 patients, 237 eyes)				
	Baseline	Follow-Up	Change	*P*-Value
Variable	Mean [SEM]	Mean [SEM]		
ETDRS (letters)	61.5 letters [0.08]	61.7 letters [0.08]	+0.2 letters	0.782
CST (μM)	329.8 μM [0.48]	303.5 μM [0.45]	−26.3 μM	<0.001
PED Height* (μM)	231.6 μM [1.87]	180.1 μM [1.91]	−51.5 μM	<0.001
Efficacy in Treatment-Naïve Patients (*N* = 37 patients, 39 eyes)				
	Baseline	Follow-Up	Change	*P*-Value
Variable	Mean [SEM]	Mean [SEM]		
ETDRS (letters)	55.8 letters [0.59]	60.7 letters [0.51]	+4.9 letters	0.076
CST (μM)	380.4 μM [2.86]	295.9 μM [2.26]	−84.5 μM	<0.001
PED Height* (μM)	199.3 μM [10.4]	105.5 μM [12.6]	−93.8 μM	0.001

This table lists the efficacy for patients after one injection of faricimab.

*if applicable.

**Fig. 1:**
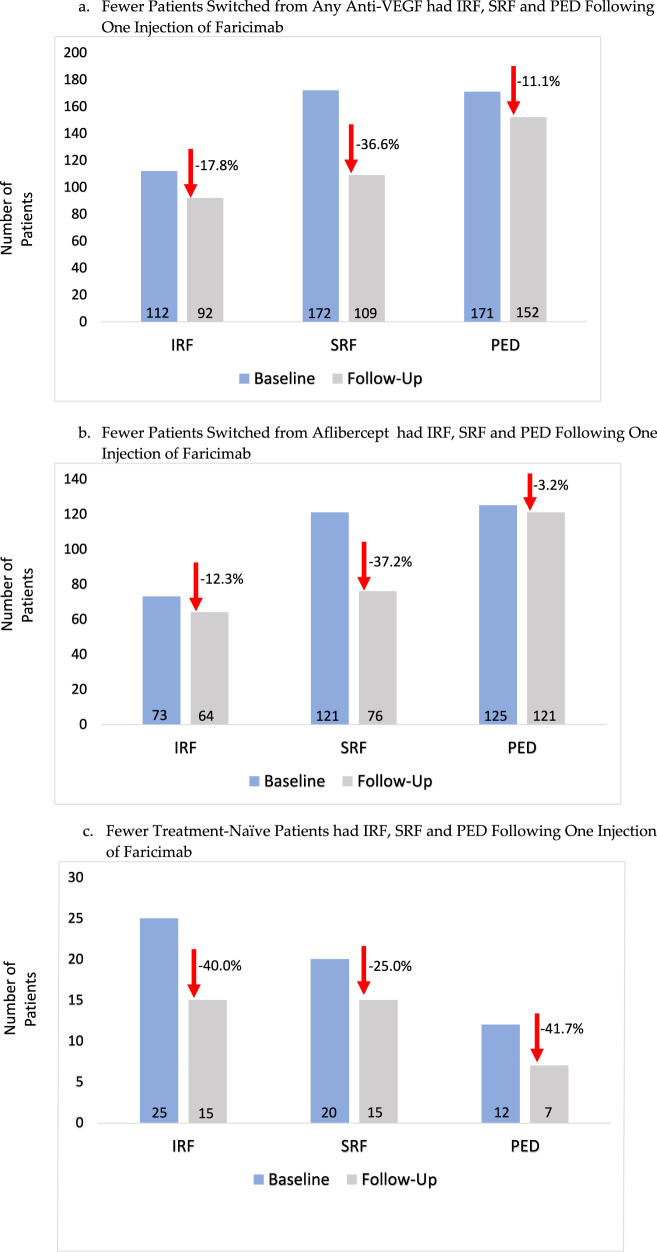
Graphs depicting the reduction rates of intraretinal fluid, subretinal fluid and pigment epithelium detachments in patients after one injection of faricimab. **a** The reduction rates of intraretinal fluid, subretinal fluid and pigment epithelium detachments in patients switched from any anti-VEGF after one injection of faricimab. **b** The reduction rates of intraretinal fluid, subretinal fluid and pigment epithelium detachments in patients switched from aflibercept after one injection of faricimab. **c** The reduction rates of intraretinal fluid, subretinal fluid and pigment epithelium detachments in treatment-naïve patients after one injection of faricimab.

Looking at anatomic outcomes based on OCT, a number of patients also demonstrated complete resolution of IRF, SRF, or PED after one injection of faricimab at a similar interval compared to prior anti-VEGF injection, as determined by investigators by lack of presence on their standard-of-care images. In patients switched from any anti-VEGF, there was a 17.8% resolution of IRF, 36.6% resolution of SRF and 11.1% resolution of PED (Fig. 1a). In patients switched from aflibercept, there was a 12.3% resolution of IRF, 37.2% resolution of SRF and 3.2% resolution of PED (Fig. 1b). In treatment-naïve patients, there was a 40.0% resolution of IRF, 25.0% resolution of SRF and 41.7% resolution of PED (Fig. 1c).

#### Follow-up after three faricimab injections

A total of 89 patients (94 eyes) completed a follow-up visit after receiving three injections of faricimab. All patients (*N* = 94 eyes) with a follow-up visit after three injections of faricimab demonstrated a mean BCVA increase of +3.4 letters (*p* = 0.03) and a mean CST reduction of −43.4 μM (*p* < 0.001) from baseline. Patients switched from any anti-VEGF (*N* = 81 eyes) demonstrated a mean BCVA increase of +2.7 letters (*p* = 0.045) and a mean CST reduction of −38.1 μM (*p* < 0.001) from baseline. Patients switched from aflibercept to faricimab (*N* = 64 eyes) demonstrated a mean BCVA increase of +2.2 letters (*p* = 0.142) and a mean CST reduction of −42.6 μM (*p* < 0.001) from baseline. Looking at previously-treated patients with BCVA improvements of 5 letters or more, 10 letters or more, or 15 letters or more after switching from any previous anti-VEGF, 24 had 5 letters or more, 17 had 10 letters or more, and 11 had 15 letters or more improvements. This data highlights the benefit of dual inhibition with faricimab in improving visual acuity in some switch patients with persistent disease activity. Treatment-naïve patients (*N* = 13 eyes) demonstrated a mean BCVA increase of +8.1 letters (*p* = 0.437) and a mean CST reduction of −80.1 μM (*p* = 0.204) from baseline. Complete data for patient efficacy after three injections of faricimab is provided in Table 3.

**Table 3:** Efficacy after Three Injections of Faricimab.

Overall Efficacy (*N* = 88 patients, 94 eyes)				
	Baseline	Follow-Up	Change	*P*-Value
Variable	Mean [SEM]	Mean [SEM]		
ETDRS (letters)	57.2 letters [0.11]	60.6 letters [0.20]	+3.4 letters	0.03
CST (μM)	359.9 μM [0.75]	316.5 μM [1.27]	−43.4 μM	<0.001
PED Height* (μM)	273.6 μM [2.66]	211.3 μM [5.63]	−62.3 μM	0.043
Efficacy in Patients Switched from Any Anti-VEGF (*N* = 75 patients, 81 eyes)				
	Baseline	Follow-Up	Change	*P*-Value
Variable	Mean [SEM]	Mean [SEM]		
ETDRS (letters)	58.2 letters [0.24]	60.9 letters [0.23]	+2.7 letters	0.045
CST (μM)	356.0 μM [1.81]	317.9 μM [1.24]	−38.1 μM	<0.001
PED Height* (μM)	277.1 μM [6.09]	213.5 μM [6.33]	−69.0 μM	0.006
	≥5 Letters	≥10 Letters	≥15 Letters	
Number of Patients with BCVA Improvements of ≥ 5, 10 or 15 Letters	24	17	11	
Efficacy in Patients Switched from Aflibercept (*N* = 61 patients, 64 eyes)				
	Baseline	Follow-Up	Change	*P*-Value
Variable	Mean [SEM]	Mean [SEM]		
ETDRS (letters)	61.0 letters [0.27]	63.2 letters [0.19]	+2.2 letters	0.142
CST (μM)	351.6 μM [1.97]	309.0 μM [1.16]	−42.6 μM	<0.001
PED Height* (μM)	248.7 μM [6.16]	191.6 μM [7.36]	−57.1 μM	0.016
Efficacy in Treatment-Naïve Patients (*N* = 13 patients, 13 eyes)				
	Baseline	Follow-Up	Change	*P*-Value
Variable	Mean [SEM]	Mean [SEM]		
ETDRS (letters)	50.5 letters [2.45]	58.6 letters [1.49]	+8.1 letters	0.437
CST (μM)	388.1 μM [11.38]	308.0 μM [11.12]	−80.1 μM	0.204
PED Height* (μM)	173.3 μM [26.68]	98.3 μM [56.77]	−75.0 μM	0.528

This table lists the efficacy for patients after three injections of faricimab.

*if applicable.

**Fig. 2:**
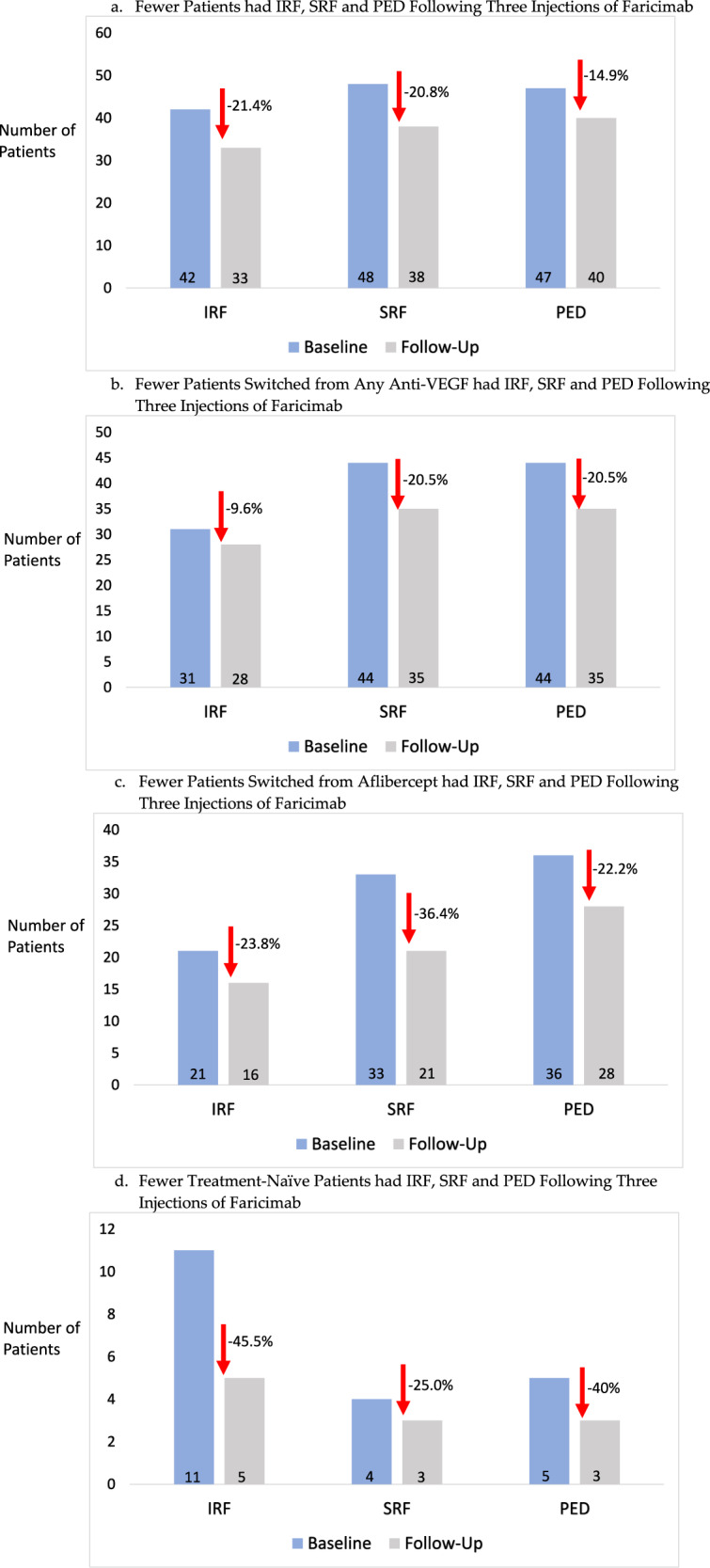
Graphs depicting the reduction rates of intraretinal fluid, subretinal fluid and pigment epithelium detachments in patients after three injections of faricimab. **a** The reduction rates of intraretinal fluid, subretinal fluid and pigment epithelium detachments in all patients after three injections of faricimab. **b** The reduction rates of intraretinal fluid, subretinal fluid and pigment epithelium detachments in patients switched from any anti-VEGF after three injections of faricimab. **c** The reduction rates of intraretinal fluid, subretinal fluid and pigment epithelium detachments in patients switched from aflibercept after three injections of faricimab. **d** The reduction rates of intraretinal fluid, subretinal fluid and pigment epithelium detachments in treatment-naïve patients after three injections of faricimab.

In all patients with follow-up after three faricimab injections, there was a 21.4% resolution of IRF, 20.8% resolution of SRF and 14.9% resolution of PED (Fig. 2a). In patients switched from anti-VEGF, there was a 9.6% resolution of IRF, 20.5% resolution of SRF and 20.5% resolution of PED (Fig. 2b). In patients switched from aflibercept to faricimab, there was a 23.8% resolution of IRF, 36.4% resolution of SRF and 22.2% resolution of PED (Fig. 2c). In treatment-naïve patients, there was a 45.5% resolution of IRF, 25.0% resolution of SRF and 40.0% resolution in occurrence of PED (Fig. 2d).

### Safety

Two cases of intraocular inflammation have been observed and reported by physicians. The first is a case of infectious endophthalmitis, treated with intravitreal antibiotics and vision returning to baseline three weeks post-treatment. The second is a case of mild anterior chamber inflammation after the fourth injection of faricimab that was treated with topical steroids until resolution, with vision returning to baseline. Widefield fluorescein angiography (FA) confirmed the absence of occlusive vasculitis. Of note, this patient has a history of anterior uveitis without occlusive vasculitis during treatment with brolucizumab, in the same eye. No cases of retinal vasculitis or retinal artery occlusion have been observed in this study. No cases of RPE tears have been reported by any investigators.

## Discussion

Registrational trials are a critical source of information on newly approved medications but practicing physicians know that an average clinical trial patient often does not enter our clinic. Real-world data is valuable for retinal physicians to understand the efficacy and safety of approved medications in patients with various demographics, comorbidities and pre-existing conditions that may not have been included in the original trials.

Positive results in both visual and anatomical parameters for patients with various treatment history are currently demonstrating the efficacy of faricimab. Particular demographics of interest are previously-treated, switch patients, especially those treated with aflibercept, the current standard-of-care treatment for many retinal physicians. These patients are not represented in the nAMD clinical trials for faricimab, resulting in a lack of data on their outcomes when treated with this novel bispecific antibody. In the presented real-world analysis, this population of patients demonstrated a maintenance of their visual acuity, along with a significant improvement in their anatomy, via CST and improvement of IRF, SRF and PED, even after just one injection. These patients continued to show improvements in these parameters after three injections of faricimab, indicating that loading doses or multiple injections may be needed to see the maximum benefit of faricimab in previously-treated patients. This data can guide physicians to optimize outcomes for their patients with persistent fluid by initially treating them with 3 monthly injections if possible, or maintaining their previous treatment interval for 3 injections before attempting to extend.

Retinal physicians currently have very efficacious, approved agents to treat nAMD with impressive safety profiles. Specialists are especially concerned about intraocular inflammation as well as drug-related retinal vasculitis and retinal artery occlusion, with some reluctant to use new treatment options without real-world evidence. A new treatment option must demonstrate comparable safety to prior standard-of-care treatment outside a clinical trial setting in the real world. At the 6 months data cut, a 0.53% incidence of intraocular inflammation has been observed with faricimab, consisting of one case of infectious endophthalmitis that may be attributed to the injection procedure itself and one case of mild anterior chamber inflammation in a patient with a history of anterior uveitis following brolucizumab injection. Although this incidence rate of inflammation is low, this data-cut timepoint is still early and inflammation is being closely monitored by all investigators.

Faricimab is the first bispecific antibody approved to treat nAMD and is the newest treatment option for the treatment of nAMD. The six months results from the TRUCKEE study with efficacy and safety data of one and three injections of faricimab highlight the benefit of faricimab in treatment naïve as well as previously-treated patients with nAMD. Future data in the TRUCKEE study will elucidate the durability of faricimab in difficult-to-treat previously-treated patients along with additional data on real-world treatment-naïve patients.

## Summary

### What was known before

- Current anti-VEGF agents are effective treatments for nAMD but do not meet the needs of all patients.
- Faricimab is being used in real-world patients with demographics different to the clinical trials.

### What this study adds

- Data on real-world patients treated with faricimab, including previously-treated high-need patients.
- Early outcomes of different populations of patients with nAMD treated with faricimab.

## Data Availability

All data generated or analysed for this data cut during this study is included in this published article.
